# Characterization of an Outbreak of Hand, Foot, and Mouth Disease in Nanchang, China in 2010

**DOI:** 10.1371/journal.pone.0025287

**Published:** 2011-09-28

**Authors:** Michelle Y. Liu, Weiyong Liu, Jun Luo, Yingle Liu, Yang Zhu, Hillary Berman, Jianguo Wu

**Affiliations:** 1 State Key Laboratory of Virology, College of Life Sciences, and Chinese-French Liver Disease Research Institute at Zhongnan Hospital, Wuhan University, Wuhan, People's Republic of China; 2 Taizhou Institute of Virology, Jiangsu Affynigen Biotechnologies, Inc., Taizhou, Jiangsu, People's Republic of China; 3 Division of Infectious Diseases and Vaccinology, School of Public Health, University of California, Berkeley, California, United States of America; 4 Wuhan Institutes of Biotechnology, Wuhan East Lake High Technology Development Zone, Wuhan, People's Republic of China; University of Nebraska Medical Center, United States of America

## Abstract

Recent outbreaks of human enterovirus 71 (EV71) infection and EV71-associated hand, foot, and mouth disease (HFMD) in China have affected millions and potentially lead to life-threatening complications in newborns. Furthermore, these outbreaks represent a significant global public health issue in the world. Understanding the epidemiology of HFMD and EV71 infection and their transmission patterns in China is essential for controlling outbreaks. However, no studies on the outbreaks of HFMD and EV71 infection in China during 2010 have been reported. In this report, we carried out an epidemiological analysis to study an outbreak of HFMD and EV71 infection in 2010 in the city of Nanchang in the Jiangxi province of People's Republic of China. From April 7 to May 11, 2010, a total of 109 HFMD cases were reported, and in this report the HFMD cases were studied by both epidemiological and laboratory analyses. The epidemiological study indicates that children aged younger than 8 years old represented more than 90% of the reported cases, with the age group of 1–3 years containing the highest number of cases. Laboratory studies detected a high prevalence of EV71 amongst the cases in our study, suggesting EV71 as a common enterovirus found in HFMD cases in Nanchang. Phylogenetic analysis of the sequence of the VP1 region of four EV71 isolates indicated that the Nanchang strains belong to the C4 subgenotype commonly found in China during outbreaks in 2008 but contain distinct variations from these strains. Our study for the first time characterizes the epidemiology of HFMD and EV71 infection in China in 2010 and furthermore, provides the first direct evidence of the genotype of EV71 circulating in Nanchang, China. Our study should facilitate the development of public health measures for the control and prevention of HFMD and EV71 infection in at-risk individuals in China.

## Introduction

Hand, foot, and mouth disease (HFMD), a common illness in children aged <10 years, is generally a benign febrile exanthematous disease but may cause potential life-threatening neurological and systemic manifestations such as encephalitis [1]. Human enterovirus 71 (EV71) and coxsackievirus A16 (CA16) are the most common causes of HFMD, although several other human enteroviruses such as coxsackievirus A4–A7, B2–B5, and enterovirus 18, can also cause the disease [2]. Clinical features of HFMD caused by these viruses are indistinguishable. The infection typically has an incubation period of 3–5 days. The main clinical manifestations are the appearance of vesicles on the palmar and plantar skin, buccal mucosa, and tongue, which may be associated with fever and lymphadenopathy [1], [2]. The oral enanthem helps to distinguish HFMD from other causes of childhood exanthems, although cases without lesions have been described [1], [3]. Uncomplicated HFMD usually resolves in 5–6 days; however, those caused by EV71 are more likely to be severe and carry a higher risk of developing neurological and cardiopulmonary complications and death [4], [5]. The predominant forms of neurological complications include aseptic meningitis and brainstem encephalitis. As a complication of systemic EV71 infection, acute cardiopulmonary failure such as pulmonary oedema has a high mortality rate [3], [6]. Understanding the epidemiology of human enteroviruses is central in controlling the infection of human enteroviruses and preventing enterovirus-associated complications including HFMD.

In the past three decades, the occurrence of HFMD cases, especially those associated with EV71, has resulted in major outbreaks throughout the world, including the US, Europe, Asia, South America, and Africa [1], [3]. The largest HFMD epidemic to date occurred in Taiwan in 1998. An estimated 1.5 million people were infected with human enteroviruses, with over 129,000 cases of HFMD or herpangina and 405 severe cases with complications including encephalitis, aseptic meningitis, pulmonary edema, hemorrhage-related acute flaccid paralysis, and myocarditis [7]. This epidemic eventually resulted in 78 deaths, most of which were due to EV71, and was followed by more outbreaks in other parts of the Asia-Pacific region, including Australia, Singapore, and Japan [8], [9], [10]. The latest large epidemic occurred in China in 2008. More than 600,000 HFMD cases and 126 deaths in children were reported from March 2008 to June 2009 [11]. At the epicenter in Anhui Province, more than 6,000 HFMD cases and 22 deaths in children were reported [11]. In addition to these large outbreaks, many areas, including Australia, Japan, Singapore, Taiwan, and Vietnam, have experienced cyclical epidemics that occur every 2–3 years [8], [9], [10]. Because of their large size and high frequency, EV71- and other enterovirus-associated HFMD epidemics have become an important public health issue in the world, especially in the Asia-Pacific region [1], [3].

As a non-enveloped virus, enteroviruses are relatively stable in the host environment as compared to enveloped viruses. The lack of a lipid envelope confers their resistance to organic solvents (e.g. ether and chloroform), alcohol, and freezing. Enteroviruses are able to survive exposure to human gastric acid, and can survive at room temperature in the environment for several days [2]. They can be detected in surface and ground water and in hot spas [12], [13]. These properties are believed to facilitate the extremely efficient transmission of human enteroviruses, even though humans are the only known natural hosts for these viruses [2]. With a RNA genome whose replication is mediated by an error-prone RNA-dependent RNA polymerase, enteroviruses mutate and evolve rapidly, leading to the generation of new viral variants [2]. Furthermore, as co-infection is common in infected individuals, existing viral strains can undergo intra- and intertypic recombination, leading to new viral variants [14], [15], [16], [17], [18]. The emergence of novel viral variants and genotypes/subgenotypes are believed to facilitate the transmission of these viruses to individuals who do not have pre-existing immunity, significantly contributing to the outbreaks of EV71 and HFMD [1], [3].

Treatment of EV71 infection and HFMD is limited as there is currently no effective chemoprophylaxis or vaccination for HFMD or EV71 infection [1], [3]. Public health-mediated strategies to block viral transmission represent the best options for controlling and preventing HFMD and EV71 infection. These strategies rely on good personal hygiene practices and social distancing measures, such as isolating the infected cases from schools and preventing further transmission of the viruses [1], [3]. The effectiveness of these measures in controlling the spread of human enteroviruses remains to be defined, and their contribution to the prevention of HFMD outbreaks needs to be further studied, especially in developing countries where sewage treatment, quality of portable water supply, and food hygiene can be suboptimal. Understanding the epidemiology of HFMD and enterovirus infection is essential for developing relevant public health measures for controlling their outbreaks.

Extensive studies have been carried out to examine the epidemiology of HFMD and enterovirus infection during the epidemic in Taiwan in 1998, as well as in outbreaks in other parts of the world including Japan, Hong Kong, the United States, and Europe [19], [20], [21], [22], [23], [24], [25], [26], [27], [28]. The epidemics of enterovirus infection in China represent a significant public health issue to the Asia-Pacific region, and potentially a global issue as a result of the efficient transmission and rapid spread of the constantly mutating and evolving viruses. Thus, it is important to examine the epidemiology of HFMD and enteroviruses and monitor their transmission patterns in China. However, little is known about the epidemiology of HFMD and EV71 infections during the epidemic in China since 2008 [11], [29], [30], [31]. No studies on the outbreaks of HFMD and enterovirus infection in China during 2010 have been reported.

**Figure 1:**
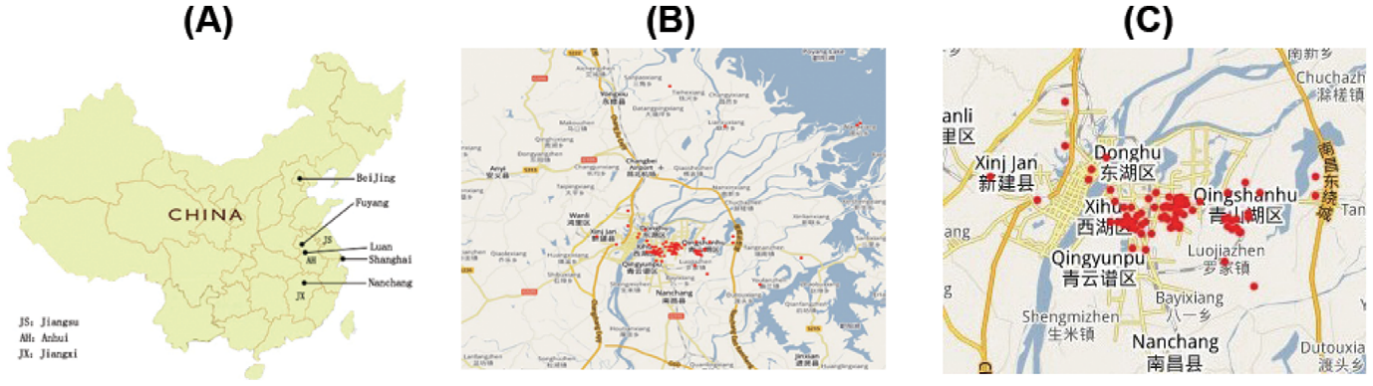
Geographic location of Nanchang in China (A) and of different HFMD cases (represented in red) in the metropolitan area (B) and city of Nanchang (C).

In this report, we studied an outbreak of HFMD and enterovirus infection in the city of Nanchang from April 7 to May 11, 2010. Nanchang is the capital city of Jiangxi Province, the southwest province adjacent to Anhui province, which was the epicenter of the 2008 HFMD epidemic (Figure 1). A total of 109 HFMD cases were reported and analyzed. Our epidemiological analyses indicate that children aged younger than 8 years old represented 90% of the reported cases, with the age group of 1–3 years containing the highest number of reported HFMD cases. Laboratory studies detected a high prevalence of EV71 amongst the cases in our study, suggesting EV71 as a common enterovirus found in HFMD cases in Nanchang. Phylogenetic analysis of the nucleotide sequences from the VP1 region of four EV71 isolates indicated that the Nanchang strains belong to the C4 subgenotype commonly found in China in 2008 but contain distinct variations from those strains. Our results reveal the epidemiological features of HFMD and EV71 infection in China in 2010 and provide insight into developing public health interventions for the control and prevention of HFMD and enterovirus in at risk individuals.

## Results

### Design of the study

During the period from April 7 to May 11, 2010, each patient who exhibited clinical symptoms of HFMD and who came or were referred to the outpatient clinic at the 9th People's Hospital of Nanchang was recorded. A case was defined as a patient who had clinical symptoms of HFMD, typically vesicles on the hand or foot, and oral lesions, with or without fever. For each patient, a thorough medical exam was given to check on other HFMD-associated symptoms, both throat swab and fecal samples were collected, and a clinical questionnaire and survey was filled out. In total, 109 cases were recorded. Based on these information and samples, two different sets of analyses were carried out. First, we carried out laboratory procedures, including conventional reverse transcription-PCR (RT-PCR) and quantitative RT-PCR (qRT-PCR), to analyze the samples for the presence of the sequences of human enteroviruses, including EV71 and CA16, which are among the most common causes of HFMD. Second, we performed epidemiological analyses with the information from the medical exams, completed questionnaires/surveys, and the laboratory results on the detection of specific viruses.

### Prevalence of human enteroviruses including EV71 and CA16 in HFMD cases in Nanchang

**Figure 2:**
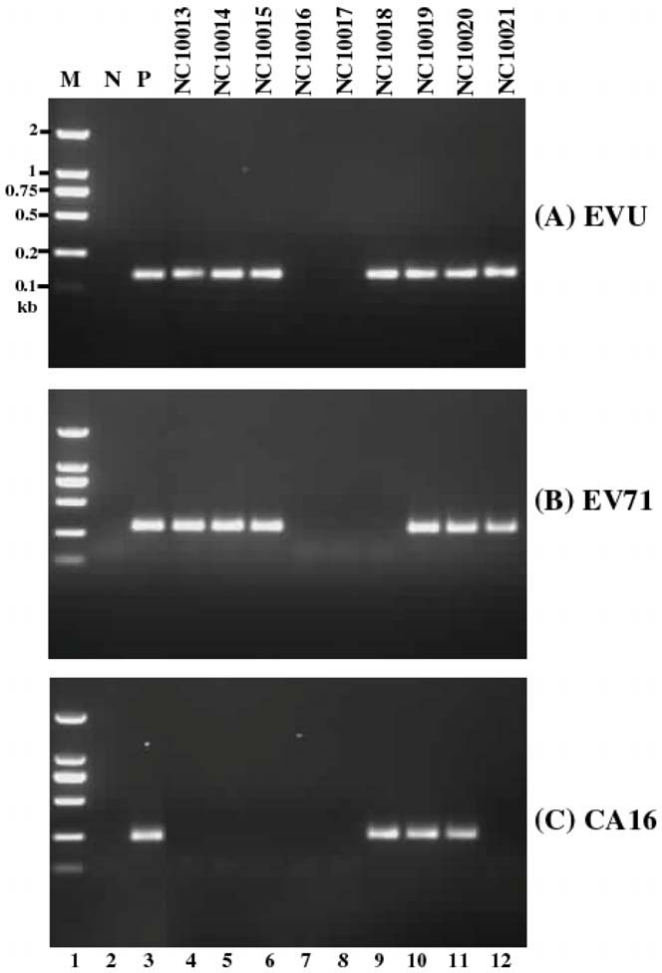
PCR reactions detecting the VP1 sequence of enterovirus 71 from samples isolated from different HFMD cases in Nanchang. RT-PCR reactions were carried out in the absence of any RNA samples (negative control)(N, lane 2) or in the presence of a positive RNA sample (P, lane 3) or RNA samples isolated from HFMD cases NC10013 (lane 4), NC10014 (lane 5), NC10015 (lane 6), NC10016 (lane 7), NC10017 (lane 8), NC10018 (lane 9), NC10019 (lane 10), NC10020 (lane 11), and NC10021 (lane 12). The amplified products were separated in 1% agarose gels. M, DL2000 DNA marker ladder (TaKaRa) (lane 1).

Three sets of primers, which specifically amplify a “universal” sequence (EVU) commonly presented in all human enteroviruses, and the specific sequences of EV71 and CA16, were used to detect the presence of human enterovirus, EV71, and CA16, respectively. Special considerations and precautions were taken to interpret the PCR results and define the presence of viruses in the sample. First, all samples were subjected to a multiplex qRT-PCR assay, which simultaneously detects the presence of human enterovirus, EV71, and CA16. Only those samples (e.g. NC10013) that were positive in both the EVU and EV71 sequence and negative in the CA16 sequence were classified as EV71 positive, while only those samples (e.g. NC10018) that were positive in both the EUV and CA16 sequence and negative in the EV71 sequence were classified as CA16 positive (Figure 2, Table S1). Second, all samples were subjected to conventional RT-PCR with each of the three sets of primers. The amplified PCR products corresponding to the common EVU of human enteroviruses, and specific sequences of EV71 and CA16 were separated on agarose gels and detected by staining (Figure 2). Only those samples (e.g. NC10013) that were positive for both the EVU and EV71 sequence and negative for the CA16 sequence were classified as EV71 positive, while only those (e.g. NC10018) positive for both the EVU and CA16 sequence and negative for the EV71 sequence were classified as CA16 positive. Third, both conventional PCR and qRT-PCR assays were carried out in duplicate for each sample and repeated three times, and only those samples with two or more positive results were considered to be positive. Fourth, only those samples that were positive in both conventional RT-PCR and qRT-PCR assays were defined to contain the respective virus. Samples with co-infection of CA16 and EV71, which were CA16 and EV71 positive, were considered EVU-positive as these samples were found to contain the EVU in our assays. Finally, to further confirm the RT-PCR and qRT-PCR results, some of the samples that were tested as positive for EV71 and CA16 were further cultured and examined for the presence of infectious viruses. EV71 viruses were isolated from the cultures, PCR products were cloned and the presence of EV71 VP1 sequence in the PCR products was verified by sequencing analysis of the cloned inserts.

Of the 109 HFMD cases, 70 (64.2%), 19 (17.4%), and 63 (57.8%) were found to contain the EVU, CA16, and EV71 sequences, respectively, suggesting that these patients were infected with an enterovirus, CA16, and EV71, respectively (Table S1). Furthermore, 12 (11.0%) were found to contain both EV71 and CA16, suggesting that these patients were co-infected with both EV71 and CA16. All the EVU-positive samples (70 cases) were also found to contain either the EV71 (63 cases or 90%) or CA16 sequence (19 cases or 27.1%) (Table S1). These results indicate that all HFMD cases in Nanchang were either infected with EV71 or CA16, and suggest that HFMD in Nanchang is more likely to be associated with EV71 infection than with CA16 infection.

### Weekly distributions of HFMD cases and incidence of viral infection

**Figure 3:**
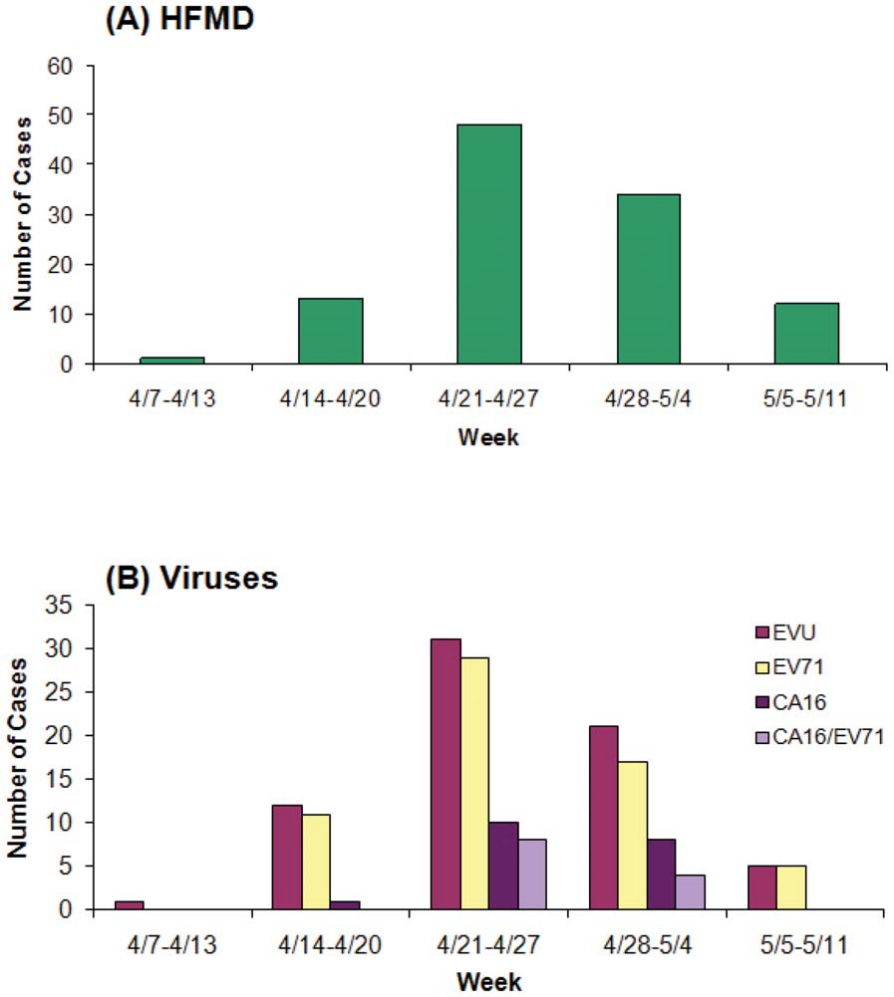
Weekly distribution of the cases of HFMD (A) and those that were tested positive in our PCR assays for enterovirus (EVU), enterovirus 71 (EV71), and coxsackievirus 16 (CA16), and positive for both EV71 and CA16 (CA16/EV71), respectively (B).

Between April 7 and May 11 of the year 2010, the 9^th^ People's Hospital of Nanchang recorded 109 suspected HFMD cases. The first reported case occurred on April 7 and the last on May 10. The outbreak in Nanchang spanned a time period of approximately 5 weeks, peaking during the week of April 21 to April 27 (Figure 3). The start and end dates of the April-May outbreak are consistent with the outbreaks in Hong Kong and Taiwan, where HFMD outbreaks occurred during late spring to early summer [3], [8]. During the following months from June to October, less than 20 HFMD cases were reported at the same hospital.

**Figure 4:**
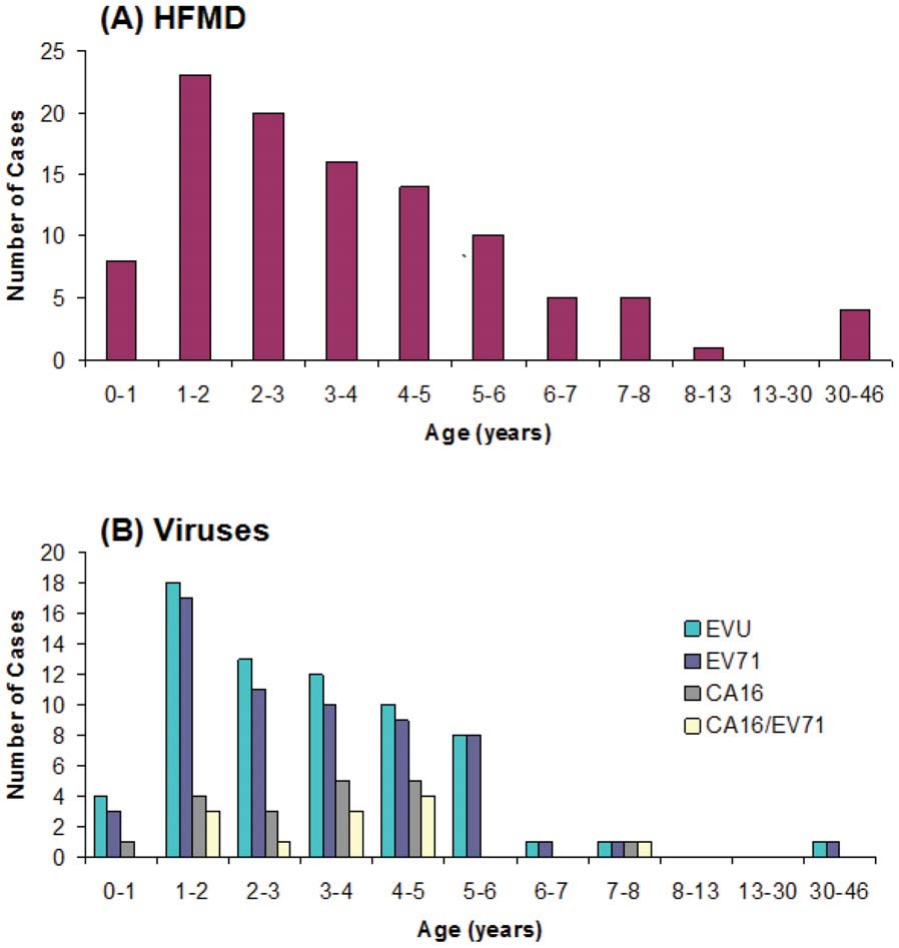
Age distribution of the cases of HFMD (A) and those that were tested positive in our PCR assays for enterovirus (EVU), enterovirus 71 (EV71), and coxsackievirus 16 (CA16), and positive for both EV71 and CA16 (CA16/EV71), respectively (B).

Amongst 109 HFMD cases, the majority (96.2%) of the cases were in children 8 years or younger, with 4 cases that were older than 30 years; the mean age being 4.37 years old. The peak of age was 1 to 2 years old (Figure 4A). When excluding cases aged greater than 8 years old, there is an overall decline of cases as the age of the patients increases. This finding is consistent with previous reports that the majority of HFMD cases are found in children aged younger than 10 years [1], [22], [29]. Furthermore, the age peak found in the HFMD outbreak of Nanchang shows that HFMD occurred most often in children of 1 year old (Figure 4A). This indicates that children of 1 year of age should in particular be targeted for HFMD control and prevention.

Of the HFMD cases, 51 cases were younger than 3 years and 55 cases were older than 3 years. Most Chinese children remain at home under the care of parents or relatives until 3 years of age and start attending preschool after that. Our results showed that 48.1% of HFMD cases were younger than 3 years old while 51.9% of cases were three years old or older (Figure 4A), suggesting that the occurrence of HFMD had little relation with schooling in Nanchang. Thus, schooling may not play a significant role in the transmission of HFMD in Nanchang.

Seventy specimens (64.2%) were determined as EVU positive, 63 specimens (57.8%) were determined as EV71 positive, 19 specimens (17.4%) were determined as CA16 positive and 12 specimens (11.0%) were determined as a co-infection of CA16 and EV71 (Table S1). The cases of EV71 infection appeared to be consistent with the pattern of the HFMD outbreak; EV71-positive cases were found to decrease as HFMD cases decreased in the age groups of 2 to 5 years old (Figure 4B). In contrast to EV71, CA16 infection does not correlate as well with HFMD infection patterns. As is evident in Figure 4B, the number of CA16 cases increased from the age groups of 2 to 4. This notion is also further supported by the prevalence (57.8% in all HFMD and 90% in all EV-positive cases, respectively) of EV71 in the patients' samples (Figure 4B). These observations suggest EV71 as the most common enterovirus found in HFMD cases and a possible cause of the HFMD outbreak in Nanchang.

### Gender distribution of HFMD cases and infection of viruses

**Figure 5:**
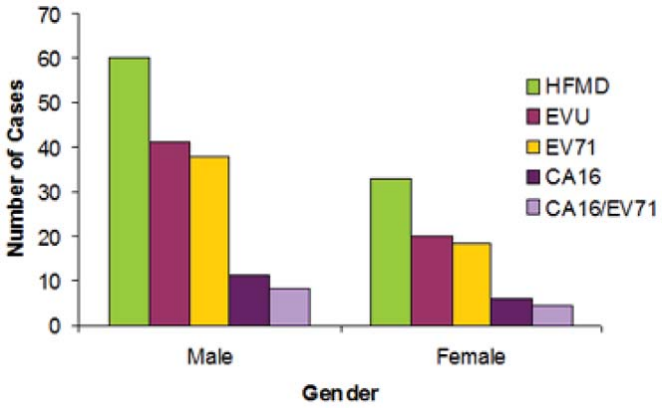
Gender distribution of the cases of HFMD and those that were tested positive in our PCR assays for enterovirus (EVU), enterovirus 71 (EV71), and coxsackievirus 16 (CA16), and positive for both EV71 and CA16 (CA16/EV71), respectively.

When excluding 6 cases lacking gender information, amongst the HFMD cases, 64.5% were boys (60 cases) and 35.5% were girls (33) (Figure 5). Of the EV71 cases, 67.9% (38) were boys and 32.1% (18) of the cases were girls (Figure 5). The number of male EV71 cases is almost twice the number of female cases.

### Symptom distribution amongst HFMD cases in Nanchang

Of the 109 cases of HFMD, only 4 cases had fever, 3 of which were the only cases requiring hospitalization (Table 1). All febrile cases were 2 years or younger. The majority of the HFMD cases in Nanchang exhibited sores in the mouth and on the hands, with a few cases of sore throat (2 cases) and loss of appetite (3 cases) (Table 1).

**Table 1:** Clinical and laboratory characteristics of different aged groups of HFMD cases in Nanchang.

Age	Under 1	1	2	3	4	5	6	7	8	Over 18
Case (No. and %)	8(7.3)	24(22.0)	20(18.3)	17(15.6)	15(13.8)	10(9.2)	5(4.6)	5(4.6)	1(0.9)	4(3.7)
EV71 (No. and %)	3(37.5)	17(70.8)	11(55.0)	11(64.7)	10(66.7)	8(80.0)	1(20.0)	1(20.0)	0(0.0)	1(25.0)
CA16 (No. and %)	1(12.5)	4(16.7)	3(15.0)	5(29.4)	5(33.3)	0(0.0)	0(0.0)	1(20.0)	0(0.0)	0(0.0)
Both EV71 and CA16 (No. and %)	0(0.0)	3(12.5)	1(5.0)	3(17.6)	4(26.7)	0(0.0)	0(0.0)	1(20.0)	0(0.0)	0(0.0)
Negative for EV71 or CA16 (No. and %)	4(50.0)	6(25.0)	7(35.0)	4(23.5)	4(26.7)	2(20.0)	4(80.0)	4(80.0)	1(100.0)	3(75.0)
Fever (No. and %)	0(0.0)	3(12.5 )	1(5.0)	0(0.0)	0(0.0)	0(0.0)	0(0.0)	0(0.0)	0(0.0)	0(0.0)
Sore throat (No. and %)	0(0.0)	2(8.3)	0(0.0)	0(0.0)	0(0.0)	0(0.0)	0(0.0)	0(0.0)	0(0.0)	0(0.0)
Loss of appetite (No. and %)	0(0.0)	2(8.3)	1(5.0)	0(0.0)	0(0.0)	0(0.0)	0(0.0)	0(0.0)	0(0.0)	0(0.0)
Oral lesion (No. and %)	8(100.0)	24(100.0)	20(100.0)	17(100.0)	15(100.0)	9(90.0)	5(100.0)	5(100.0)	1(100.0)	4(100.0)
Vesicles on hand and foot (No. and %)	8(100.0)	24(100.0)	20(100.0)	16(94.1)	15(100.0)	9(90.0)	5(100.0)	5(100.0)	1(100.0)	4(100.0)

### Geographical distribution of HFMD cases in Nanchang

Of all 109 reported HFMD cases, 52 (47.7%) were found in the Qingshanhu district, suggesting that the transmission patterns of those cases were unique and different from other districts in Nanchang (Table 2, Table 3). The majority of the cases in the Qingshanhu district were caused by EV71 (Table 2, Table 3). Further analysis of the geospatial distribution within the Qingshanhu district revealed two HFMD case clusters: one in the Nanchang Steel Mill Industrial Park and the other in the Jiangxi Tire Manufacturing Industrial Park (Figure 1C). For further investigation of these two clusters, field trips were made to both areas. Both Industrial Parks contain manufacturing factories, community complexes, shopping and business districts and schools.

**Table 2:** Distribution of the numbers of HFMD cases in different districts of Nanchang.

District	HFMD Cases
Donghu	5
Fuzhou	1
Gan	1
Honggutan	3
Nanchang	8
Qingshanhu	52
Qingyunpu	5
Xihu	17
Xinjian	9

**Table 3:** Distribution of the incidences of viral infections in different districts of Nanchang.

District	EVU	EV71	CA16	CA16/EV71
Donghu	2	1	1	0
Fuzhou	0	0	0	0
Gan	0	0	0	0
Honggutu	3	3	0	0
Nanchang	2	2	1	1
Qingshanhu	38	33	14	9
Qingyunpu	5	5	2	2
Xihu	9	9	0	0
Xinjian	6	5	1	0

### Molecular characterization of EV71 strains in Nanchang

**Figure 6:**
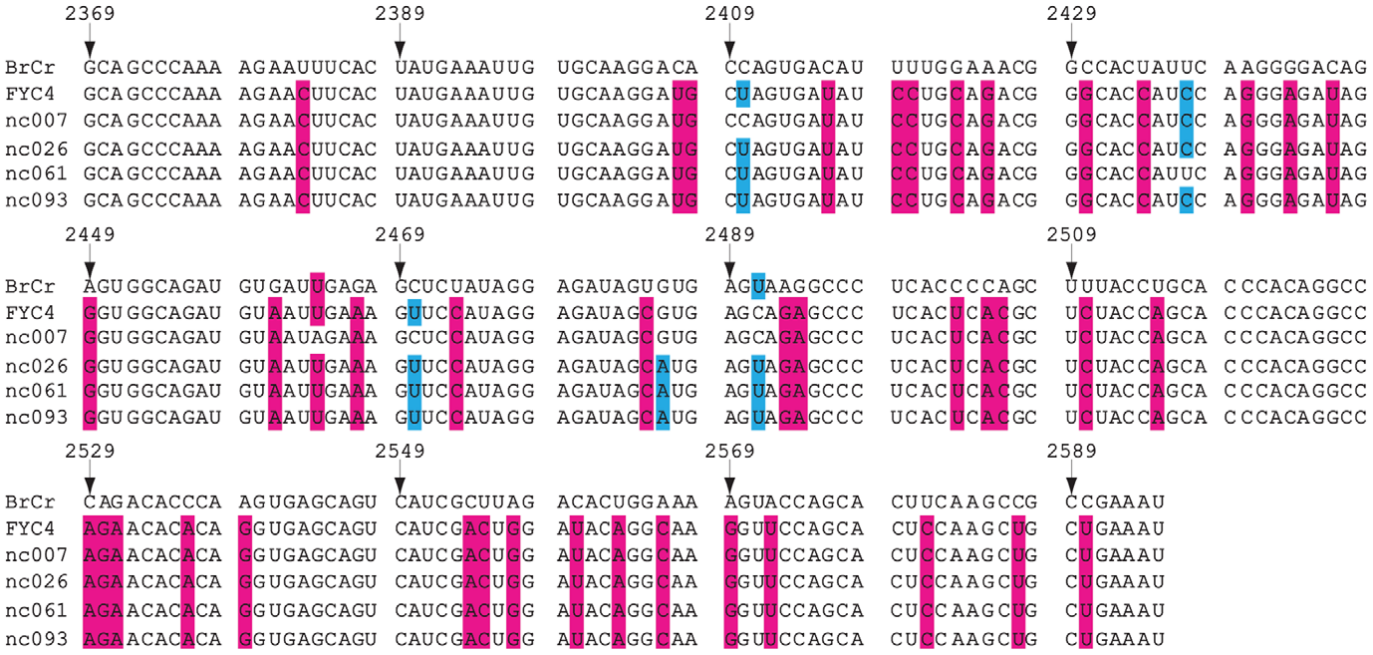
Nucleotide sequence comparison of a part of the VP1 genes between four EV71 strains isolated in Nanchang and the reference viruses EV71-BrCr (BrCr) and EV71-FYC4 (FYC4). The nucleotides that are not identical among all the virus sequences are highlighted. Numbering starts at the 5′ terminus of the EV71 genome.

**Figure 7:**
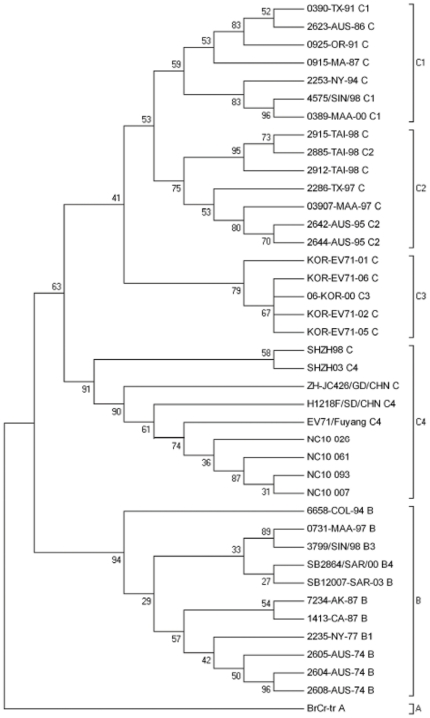
Phylogenetic analysis of the four EV71 isolates from Nanchang and the reference viruses. The evolutionary relationships among these viruses were estimated by the method of the neighbor-joining method with 1000 bootstraps. Sequence comparisons to the reference viruses were performed by a multiple alignment of the nucleotide sequences of the VP1 region.

To characterize the EV71 strains circulating in Nanchang and investigate their genetic origin, we isolated viruses from four EV71-positive samples, which represent different geographic regions of Nanchang and patients from different age groups, by culturing them in rhabdomyosarcoma (RD) cells. The isolation of EV71 viruses from these samples further confirms our PCR results. The isolated viruses will be used for further analysis of the biological properties of the EV71 viruses circulating in Nanchang. The VP1 regions of these four EV71 isolates were amplified by PCR, cloned, and sequenced. Figure 6 shows the alignment of the nucleotide sequence of the VP1 region of the four EV71 isolates from Nanchang and the reference strains EV71-BrCr (a genotype A strain that was isolated in California in 1968) and EV71-FYC4 (a genotype C4 strain that was isolated in Fuyang, Anhui of China in 2008) obtained from GenBank, using the Clustal W program [32]. Using the neighbor-joining algorithm in MEGA software, we constructed a phylogenetic tree with the sequences of these four isolates and 36 reference EV71 strains from Genbank [33] (Figure 7). The reference viruses consisted of EV71 strains isolated from different parts of the world during the last four decades, and can be classified into three large groups corresponding to genotypes A, B, and C. The phylogenetic analysis revealed that the Nanchang isolates are grouped into the same cluster of genotype C, and have a close evolutionary relationship with EV71 strains of the C4 subgenotype lineage. EV71 strains of the C4 subgenotype lineage have been commonly found in China during the EV71 outbreaks in 2008 [11], [29], [31] and are distinct from those virus strains circulating in Australia, Europe, and US. Indeed, all four Nanchang sequences cluster well with the VP1 sequences of the EV71 viruses recently found in China (within the C4 subgenotype lineage) but not those EV71 strains circulating in the rest of the world such as Australia, Europe, and US (Figure 7). This result indicates that these Nanchang EV71 strains belong to the subgenotype C4 and may have originated from adjacent provinces in China such as Anhui, the epicenter of EV71 outbreaks in 2008.

## Discussion

In this study, we have carried out an epidemiological analysis to study an outbreak of HFMD and human enterovirus infection in Nanchang, China between April 7 and May 11, 2010. Our results provide direct evidence that children aged 8 years old or younger represented more than 90% of the reported cases, with the age group of 1–3 years containing the highest number of HFMD and EV71-positive cases. Seventy samples were positive for human enteroviruses, 63 which contained EV71 and 19 of which contained CA16. Our study represents the first to investigate the epidemiology of HFMD and EV71 infection during outbreaks in China in 2010.

It is possible that the epidemiological analysis carried out in this study may not truly represent the transmission and distribution of HFMD and viral infections during outbreaks as data collection may have been artificial and partial, and the laboratory analyses may have contained false positive or false negative results generated in the RT-PCR and qRT-PCR assays. To address these issues, we have carefully designed the study and have taken special precautions to interpret the results. First, all clinical data were obtained in the 9^th^ People's Hospital of Nanchang, which is the only regional hospital in the city specializing in infectious diseases. As such, most of the patients that were suspected of HFMD and seen in other hospitals in the city, such as the Children's Hospital of Nanchang, were referred to the 9^th^ Hospital. Second, each patient was given a thorough medical exam, throat swab and fecal samples were collected, and a clinical questionnaire and survey was filled out. Third, to prevent false positive and negative results in the laboratory tests, each sample was subjected to both conventional PCR and qRT-PCR assays. For each assay, three sets of primers amplifying a common “universal” sequence of enterovirus and specific sequences of EV71 and CA16 were used to specifically detect the presence of human enterovirus, EV71, and CA16, respectively. Each assay was performed in duplicate and repeated three times, and only those samples that were tested positive consistently in both assays were defined as positive for the viruses. Finally, to further confirm the RT-PCR and qRT-PCR results, some of the samples that were tested positive for EV71 and CA16 were further cultured and examined for the presence of infectious viruses. Thus, we believe that the results presented in this study may accurately represent the epidemiology of HFMD and enteroviruses in the patient population in Nanchang in 2010.

To characterize the EV71 strains circulating in Nanchang, we isolated viruses from the four EV71-positive samples that were used for sequencing analysis of the VP1 region, by culturing them in rhabdomyosarcoma (RD) cells. These four samples were from patients representing different geographic regions of Nanchang and from different age groups. The isolation of EV71 viruses from these samples further confirms our PCR results, and will be used for further analysis of the biological properties of the EV71 viruses circulating in Nanchang. We note that all our HFMD cases did not exhibit severe neurological and cardiopulmonary complications. Further investigation of the HFMD cases in China, including the isolation and characterization of the viruses from those cases exhibiting neurological and cardiopulmonary complications, will help reveal the correlation between EV71 infection and severe HFMD-associated complications.

It is generally believed that enteroviruses transmit among individuals via intra-familial and intra-community routes including attendance in schools [34], [35]. Previous studies have indicated that intra-familial transmission occurs commonly [35]. In a prospective cohort, the rate of symptomatic infection after household transmission is higher than that in other community settings, possibly because of more prolonged contact with the cases and a larger infective dose [35]. Furthermore, transmission rates from infected children to siblings were as high as 84% [35]. Our results are consistent with these observations. Most of the children 1 year old or younger did not attend any day care schools and were instead cared for at home by family members, primarily parents and relatives in our study based on our telephone interview survey. Most of these children never had contact with people outside of their immediate household members, strongly suggesting that they contracted the disease and enterovirus from family members. Due to the current one child per family policy in China, it is difficult to assess the transmission from infected children to siblings within a household. Our results suggest that asymptomatic adult carriers of human enteroviruses such as EV71 and CA16 may represent a major reservoir for the transmission of these viruses in a household setting, although we can not rule out other possible unidentified means of transmission within the households of these affected children. Little is currently known about the level of viral shedding from these adult carriers. Our results suggest that screening of asymptomatic adult carriers may be necessary in order to prevent the transmission of HFMD and EV71 to children 1 year old or younger, who may be most at-risk to infection.

Our preliminary genotype analysis primarily focused on the viruses isolated from the EV71-positive samples. EV71 has been shown to cause more severe complications in HFMD patients than CA16 and several EV71 subtypes associated with different clinical manifestations have been reported, while CA16 appears to be more homogenous and have fewer subtypes than EV71 and is much less associated with severe HFMD-associated complications than EV71 [1], [2]. It will be interesting to determine the genotype of the CA16 virus isolates from our HFMD samples. Meanwhile, it is difficult to determine the statistical difference among the study groups in the current study, possibly due to the small size of the samples and the complexity of the issues between HFMD and EV71/CA16 infections. It will be important to carry out detailed statistical analyses on a larger sample size and these analyses will further elucidate the correlation between EV71/CA16 infections and HFMD-associated diseases.

In contrast to previous epidemics in Taiwan in 1998 and in the Anhui Province of China in 2008, most of our cases are benign and none of our cases was severe or associated with neurological complications. This is surprising since a substantial number of cases were infected by EV71, which usually induces a much more severe illness than coxsackievirus A. One possibility is the unique susceptibility of individuals in Nanchang to enterovirus strains. It is also possible that the viral strains in Nanchang are attenuated and different from those circulating in other epidemic regions including the Anhui Province. No cases of HFMD or EV71 infection in the Nanchang area have been previously reported. Our results in this study provide the first direct evidence that four EV71 strains isolated from the HFMD cases in Nanchang are genotype C4 viruses. Phylogenetic analyses suggest that these isolates may be closely related to those circulating in adjacent Chinese provinces such as Anhui Province but contain distinct mutations at the VP1 region which sequences have been determined. It is currently unknown whether these mutations may contribute to the attenuated complications associated with the patients infected with these isolates. It is also unclear whether these isolates also contain mutations at other regions of the viral genome. Determination of the genomic sequence of these strains and characterization on their ability to replicate and cause pathogenesis will clarify these important issues. Further studies on these important issues will facilitate the control and prevention of the infections of human enteroviruses and their associated diseases including HFMD.

## Materials and Methods

### Ethics Statement

All research involving human participants was approved by the Institutional Review Board of the College of Life Sciences, Wuhan University (Wuhan, China), leaded by Dr. Hongbing Shu, the Dean of the college, in accordance with the guidelines for the protection of human subjects. Written informed consent was obtained from each participant. No research involving human participants was carried out at the University of California-Berkeley.

### Clinical information and specimen collection

All reported HFMD cases in Nanchang were recorded by medical practitioners of the 9^th^ People's Hospital of Nanchang. All cases were used in this study. Cases consisted of children and adults living within the districts of Nanchang, Jiangxi province. HFMD patients were diagnosed mainly by observation of the presence of hand or mouth blisters. In many cases, the parents of the patients noticed the sores and, out of worry, sent their children to the hospital to check for HFMD. Documentation of patients' name, age, symptoms and contact information was performed by doctors of the 9^th^ People's Hospital of Nanchang. Hospital scientists then used the documents to organize and fill the mentioned information into clinical surveys conducted by the hospital. These surveys were collected from the hospital and used in this research project for an epidemiologic study. Throat swabs and fecal samples were also collected from each case when applicable by medical practitioners of the 9^th^ People's Hospital of Nanchang. These samples were used in the lab for viral isolation and diagnosis. Several cases had visited the smaller hospitals within their district area before being referred to the 9^th^ People's Hospital of Nanchang.

### Telephone interviews and site visits

Telephone interviews were carried out in order to obtain additional information about patients' activities during and prior to their infected period. Telephone numbers were used from the contact information collected from the clinical surveys. Parents of the patients were contacted and questioned regarding the activities of their child and other details. Responses were then collected and stored in a survey. In addition, two trips were made, in the August and December of 2010, to Nanchang by car to the 9^th^ People's Hospital of Nanchang and preschools and Chengdong primary school in the industrial park areas where there occurred clusters of HFMD cases. Playgrounds and classrooms were observed in all of the schools. Sleeping areas were also observed in the preschools. Teachers were interviewed regarding childrens' activities during school, the school's prevention methods towards HFMD and information known about the cases that occurred there. In addition, the scientists responsible for creating and filling in the clinical surveys were interviewed at the 9^th^ People's Hospital of Nanchang.

### Viral RNA extraction from specimens and reverse transcription

Viral RNA was extracted from the throat and fecal samples received from the 9^th^ People's Hospital of Nanchang using a Roche High Pure Viral RNA Extraction Kit (Roche, Germany), according to the manufacturer's experimental protocols. Reverse transcription was carried out in the presence of M-MLV Reverse Transcriptase (Promega, USA), following the procedure provided by Promega, Inc. Briefly, the following components were added to a sterile RNase-free microcentrifuge tube in a reaction volume of 50 µl that contained 50 mM Tris-HCl (pH 8.3), 75 mM KCl, 3 mM MgCl_2_, 10 mM DTT, 200 unit M-MLV RT enzyme (Promega, Inc.), 25 unit RNAsin (Promega, Inc.), 0.1 mM dATP, 0.1 mM dCTP, 0.1 mM dGTP, 0.1 mM dTTP, and the template RNA samples. The reaction components were mixed gently and incubated for 60 minutes at 37°C.

### Conventional PCR analysis

Both conventional PCR and quantitative real-time PCR were performed in this study to detect the presence of the common (universal) sequence of enterovirus (EVU), and the specific sequences of enterovirus 71 (EV71) and coxsackievirus 16 (CA16). Samples with co-infection of CA16 and EV71, which were CA16 and EV71 positive, were considered as EVU-positive as these samples were positive for the presence of EVU in our assays. The conventional PCR mix (in a reaction volume of 25 µl) contained the following: 10 mM Tris-HCl, pH 8.8, 50 mM KCl, 0.08% (v/v) Nonidet P40, 1.5 mM MgCl_2_, 50 µM dATP, 50 µM dCTP, 50 µM dGTP, 50 µM dTTP, 0.4 µM forward primer, 0.4 µM reverse primer, 1 U Taq DNA polymerase (Fermentas, Shenzhen, China), and the cDNA template samples. Each sample was tested individually for the presence of the common (universal) EV sequence (EVU), and the specific sequences of EV71 and CA16. The forward and reverse primers for detection of the universal EV sequence (EVU) is PE2 (5′-tccggcccctgaatgcggctaatcc-3′) and PE1 (5′-acacggacacccaatagtcggtcc-3′), respectively. The forward and reverse primers for detection of the specific EV71 sequence (EV71) is EV71-s (5′-gcagcccaaaagaatcac-3′) and EV71-a (5′-atttcagcagcttggagtgc-3′), respectively. The forward and reverse primers for detection of the specific CA16 sequence (CA16) is CA16-s (5′-attggtgctcccactacagc-3′) and CA16-a (5′-tcagtgttggcagctgtagg-3′), respectively. The conventional PCR reactions were performed in a *Veriti 96 Well thermal Cycler* PCR Instrument (Applied Biosystems, USA) under the following conditions: 95°C for 3 min and 33 cycles of 95°C for 30 seconds, 50°C for 30 seconds, and 72°C for 40 seconds, followed by a final 72°C for 7 min. The amplified PCR products of the universal EV (EVU) (116 bp), EV71 (226 bp), and CA16 sequences (208 bp) were separated on 1.5% agarose gels and stained with Gelred and methyl blue.

### Quantitative RT-PCR analysis

One-step real-time RT-PCR assays using FAM and VIC were conducted to detect the presence of the universal EV (EVU) sequence, and the specific EV71 and CA16 sequences in each clinical sample. Samples with co-infection of CA16 and EV71, which were CA16 and EV71 positive, were considered EVU-positive as these samples were found to contain the EVU in our assays. Reactions were prepared in a 25-µL volume by using a One Step PrimeScript RT-PCR kit (BioPerfctus Technologies, Inc., Shanghai, China) and performed in a Light Cycler 480 II (Roche, Germany) using two different wavelength channels: FAM and VIC. FAM detects the presence of EVU and CA16 while VIC detects the presence of EV71, according to the protocols from BioPerfctus Technologies, Inc. (Shanghai, China). For each sample, one reaction was set up to detect EV71 and CA16 while another reaction was set up to detect the EVU sequence. All the reactions were performed in duplicate.

### Sequencing and phylogenetic analyses

The amplified products from the conventional PCR reactions were cloned into the pMD-18T vector (TaKaRA), and subsequently subjected to bi-directional DNA sequencing in Invitrogen, Inc. (Shanghai, China). The Genbank accession numbers of the VP1 sequences of the four EV71 isolates reported in this study are JF892540-892543. Nucleotide BLASTn analysis (http://www.ncbi.nlm.nih.gov/BLAST) was used to identify related reference viruses, and the reference sequences were obtained from GenBank. Pair-wise sequence alignments were performed with the Megalign program (DNASTAR) to determine nucleotide and amino acid sequence similarities. Those newly identified nucleotide sequences were aligned with EV71 VP1 reference sequences and sequences previously found in China, using Clustal W program implemented with MEGA 4.0 [32], [33]. To understand the evolutionary characterization of EV71 viruses isolated in this study, phylogenetic analyses of the aligned sequences for a part of the VP1 gene were performed by the neighbor-joining method with 1000 bootstraps using MEGA 4.0.

### Data analysis

Information contained in the clinical surveys collected from the 9^th^ People's Hospital of Nanchang were stored and filled into new surveys using the data analysis program *Epi Info* (http://wwwn.cdc.gov/epiinfo/). Data analysis was performed by calculating the frequencies of the different variables within the surveys. For certain variables some cases were omitted due to missing information included on the corresponding surveys.

## Supporting Information

Table S1 **Detection of the presence of a human enterovirus, EV71, and CA16 in the HFMD samples by quantitative real-time RT-PCR.**
(DOC)
