# Supplementary material for: Characterization of an Outbreak of Hand, Foot, and Mouth Disease in Nanchang, China in 2010
Source: PLoS One. 2011 Sep 28;6(9):e25287. doi: 10.1371/journal.pone.0025287 (PMC3182205; doi:10.1371/journal.pone.0025287)
Supplement: Table S1 — Detection of the presence of a human enterovirus, EV71, and CA16 in the HFMD samples by quantitative real-time RT-PCR. (DOC) [file pone.0025287.s001.doc]

**SUPPORTING INFORMATION**

Table S1. Detection of the presence of a human enterovirus, EV71, and CA16 in the HFMD samples by quantitative real-time RT-PCR

| Number | EVU | CA16 | EV71 |
| --- | --- | --- | --- |
| NC10001 | positive | ——^a^ | positive |
| NC10002 | —— | —— | —— |
| NC10003 | positive | —— | positive |
| NC10004 | positive | Positive | —— |
| NC10005 | positive | —— | positive |
| NC10006 | positive | —— | positive |
| NC10007 | positive | —— | positive |
| NC10008 | positive | —— | positive |
| NC10009 | positive | —— | positive |
| NC10010 | positive | —— | positive |
| NC10013 | positive | —— | positive |
| NC10014 | positive | —— | positive |
| NC10015 | positive | —— | positive |
| NC10016 | —— | —— | —— |
| NC10017 | —— | —— | —— |
| NC10018 | positive | positive | —— |
| NC10019 | positive | positive | positive |
| NC10020 | positive | positive | positive |
| NC10021 | positive | —— | positive |
| NC10022 | positive | positive | positive |
| NC10023 | positive | —— | positive |
| NC10024 | positive | positive | positive |
| NC10025 | positive | —— | positive |
| NC10026 | positive | —— | positive |
| NC10027 | positive | —— | positive |
| NC10028 | positive | positive | positive |
| NC10029 | positive | —— | positive |
| NC10030 | positive | —— | positive |
| NC10031 | positive | positive | positive |
| NC10032 | —— | —— | —— |
| NC10033 | —— | —— | —— |
| NC10034 | —— | —— | —— |
| NC10035 | —— | —— | —— |
| NC10036 | positive | —— | positive |
| NC10037 | —— | —— | —— |
| NC10038 | —— | —— | —— |
| NC10039 | positive | —— | positive |
| NC10040 | positive | —— | positive |
| NC10041 | —— | —— | —— |
| NC10042 | positive | —— | positive |
| NC10043 | positive | —— | positive |
| NC10044 | —— | —— | —— |
| NC10045 | positive | positive | positive |
| NC10046 | positive | positive | positive |
| NC10047 | positive | —— | positive |
| NC10048 | positive | —— | positive |
| NC10049 | positive | —— | positive |
| NC10050 | —— | —— | —— |
| NC10051 | —— | —— | —— |
| NC10052 | positive | —— | positive |
| NC10053 | positive | —— | positive |
| NC10054 | positive | —— | positive |
| NC10055 | positive | positive | —— |
| NC10056 | —— | —— | —— |
| NC10057 | —— | —— | —— |
| NC10058 | —— | —— | —— |
| NC10059 | positive | —— | positive |
| NC10060 | positive | —— | positive |
| NC10061 | positive | —— | positive |
| NC10062 | positive | —— | positive |
| NC10063 | positive | —— | positive |
| NC10064 | —— | —— | —— |
| NC10065 | —— | —— | —— |
| NC10066 | —— | —— | —— |
| NC10067 | —— | —— | —— |
| NC10068 | positive | positive | positive |
| NC10069 | positive | positive | positive |
| NC10070 | positive | positive | —— |
| NC10071 | positive | positive | positive |
| NC10072 | positive | positive | positive |
| NC10073 | positive | —— | positive |
| NC10074 | —— | —— | —— |
| NC10075 | —— | —— | —— |
| NC10076 | —— | —— | —— |
| NC10077 | positive | —— | positive |
| NC10078 | positive | —— | positive |
| NC10079 | positive | —— | positive |
| NC10080 | positive | —— | positive |
| NC10081 | —— | —— | —— |
| NC10082 | positive | positive | —— |
| NC10083 | positive | positive | —— |
| NC10084 | —— | —— | —— |
| NC10085 | positive | —— | positive |
| NC10086 | —— | —— | —— |
| NC10087 | positive | —— | positive |
| NC10088 | —— | —— | —— |
| NC10089 | positive | —— | positive |
| NC10090 | —— | —— | —— |
| NC10091 | positive | positive | —— |
| NC10092 | positive | —— | positive |
| NC10093 | positive | —— | positive |
| NC10094 | positive | —— | positive |
| NC10095 | —— | —— | —— |
| NC10096 | positive | —— | positive |
| NC10097 | —— | —— | —— |
| NC10098 | —— | —— | —— |
| NC10099 | positive | —— | positive |
| NC10100 | positive | —— | positive |
| NC10101 | positive | —— | positive |
| NC10102 | —— | —— | —— |
| NC10103 | positive | —— | positive |
| NC10104 | positive | —— | positive |
| NC10105 | —— | —— | —— |
| NC10106 | —— | —— | —— |
| NC10107 | —— | —— | —— |
| NC10108 | —— | —— | —— |
| NC10111 | —— | —— | —— |
| NC10112 | —— | —— | —— |
| NC10113 | —— | —— | —— |

^a^ Negative
